# Algorithmic differentiation for plane-wave DFT: materials design, error control and learning model parameters

**DOI:** 10.1038/s41524-025-01880-3

**Published:** 2025-12-04

**Authors:** Niklas Frederik Schmitz, Bruno Ploumhans, Michael F. Herbst

**Affiliations:** 1 Mathematics for Materials Modelling (MatMat), Institute of Mathematics & Institute of Materials, École Polytechnique Fédérale de Lausanne, Lausanne, Switzerland; 2 National Centre for Computational Design and Discovery of Novel Materials (MARVEL), École Polytechnique Fédérale de Lausanne, Lausanne, Switzerland

**Keywords:** Chemistry, Materials science, Mathematics and computing, Physics

## Abstract

We present a differentiation framework for plane-wave density-functional theory (DFT) that combines the strengths of forward-mode algorithmic differentiation (AD) and density-functional perturbation theory (DFPT). In the resulting AD-DFPT framework derivatives of any DFT output quantity with respect to any input parameter (e.g., geometry, density functional or pseudopotential) can be computed accurately without deriving gradient expressions by hand. We implement AD-DFPT into the Density-Functional ToolKit (DFTK) and show its broad applicability. Amongst others we consider the inverse design of a semiconductor band gap, the learning of exchange-correlation functional parameters, or the propagation of DFT parameter uncertainties to relaxed structures. These examples demonstrate a number of promising research avenues opened by gradient-driven workflows in first-principles materials modeling.

## Introduction

The central goal of first-principles modeling is to provide access to accurate predictions of atomistic properties. Considering the most widely used approach, density-functional theory (DFT)^1,2^, properties are commonly obtained from the response of the electronic structure to an external perturbation, i.e., as derivatives of DFT simulation outcomes. For example, phonon dispersion relations can be obtained from the second-order derivative of the DFT energy wrt. atomic positions^3^, or dielectric susceptibility as the derivative of polarization wrt. electric field strength^4^.

Density-functional perturbation theory (DFPT)^3^ provides the rigorous framework to compute DFT derivatives. It has taken decades of joint community effort to iron out many subtleties of DFT gradient computation^5–12^, resulting in the efficient implementations available nowadays in widespread DFT codes^13–17^. However, the generality of these implementations varies, as they are typically limited to specific types of DFT functionals and perturbations. Manually adding support for additional classes of DFT models or DFT derivatives can be a daunting task, resulting in a substantial obstacle to rapid exploration of novel avenues in materials modeling.

In an attempt to avoid this considerable human effort, researchers often have to resort to finite-difference-based approaches, see for example the comprehensive frameworks for DFT elasticity tensors^18,19^, phonons^20^, or other spectroscopic properties^21^. However, finite-difference techniques suffer from well-known deficiencies, such as their sensitivity to numerical noise and the need to find an appropriate step size^10,18^.

This article explores the computation of DFT gradients employing algorithmic differentiation (AD) techniques. AD offers a rigorous mathematical approach to compute derivatives automatically and accurately^22,23^. Recent advances in general-purpose AD systems^24–27^ have significantly broadened their applicability in scientific computing^28,29^. As we will discuss, AD does not replace DFPT; rather, it allows to set up and solve appropriate DFPT problems transparently. This results in a powerful systematic AD-DFPT framework capable of computing gradients in an end-to-end fashion, across entire DFT workflows.

The use of AD techniques in atomistic modeling is hardly a novelty; see the broad list of examples related to machine-learning force fields^30–33^, the differentiable programming of thermodynamical observables^34–37^, or model Hamiltonians^38–40^. Considering differentiable DFT simulations specifically, first software packages^41–44^ with AD capabilities have recently appeared for simulations employing Gaussian basis sets. Their ability to compute arbitrary DFT gradients has already enabled novel approaches to machine-learned DFT functionals or the inverse design of molecules^41,45–47^. In plane-wave DFT-related settings, recent work focused on AD in orbital-free DFT^48^ or for implementing direct minimization algorithms^49^.

However, a systematic AD treatment in standard plane-wave DFT and DFPT has so far remained an open challenge. The underlying difficulty stems from the distinct mathematical structure of plane-wave methods: While Gaussian basis sets result in tractable dense matrices for the Hamiltonian or the density matrix, for plane-wave basis sets, these central objects are much larger, but structured. Exploiting this structure appropriately—e.g., via fast Fourier transforms (FFTs) and iterative algorithms—is essential to obtain an efficient code^17^. The same strategies are indispensable when computing DFT derivatives. In other words, the successful use of AD in the plane-wave setting requires the careful incorporation of the algorithmic insights that underlie decades of DFPT development.

**Fig. 1:**
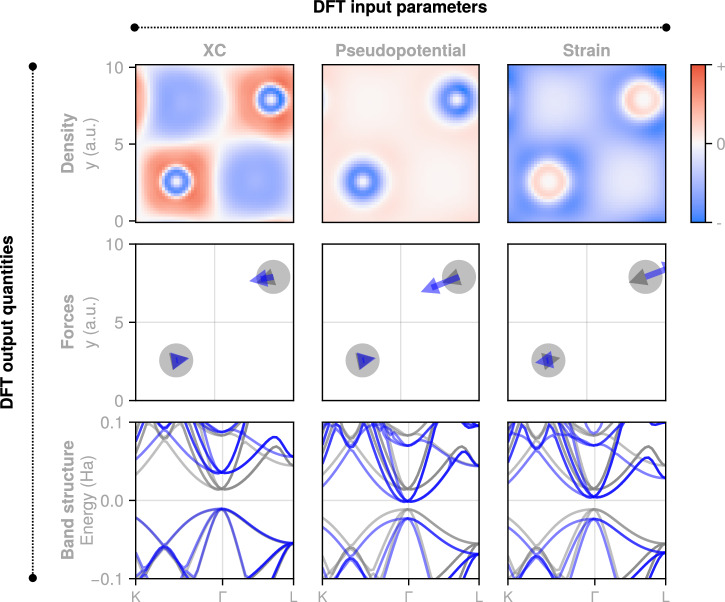
Systematic DFT derivatives. Examples of physical quantities (rows) differentiated with respect to input parameters (columns), illustrating the combinatorial range of quantity-parameter derivatives readily accessible with our AD-DFPT framework. Quantities are displayed for a silicon unit cell. Densities and non-zero forces are shown along a *z* = 0 plane and the structure was slightly distorted. The parameter-induced changes have been scaled to improve visibility.

In this work, we present a first AD-DFPT framework for plane-wave DFT. At the conceptual level, our approach integrates AD with DFPT transparently and with high generality: geometric parameters such as strain, and DFT model parameters such as those of the exchange-correlation (XC) functional or pseudopotentials (PSPs) now all enter on an equal footing; see Fig. 1. The combinatorial number of possible derivatives of DFT output quantities versus any of these parameters thus become readily available for use in materials modeling.

Our framework uses forward-mode AD^22^, where perturbations are propagated forward from input parameters to all outputs, thus providing a natural generalization of traditional DFPT. Computationally, the complexity of accumulating explicit derivative tensors (or gradients) depends on the number of independent input perturbations, just like in traditional DFPT. The extension of AD-DFPT to reverse-mode AD, suitable for high-dimensional gradients arising from loss functions of many input parameters (such as training highly parametrized machine-learned XC functionals), is left as a promising avenue for future extension.

Practically, we realized our AD-DFPT framework by direct implementation into the density-functional toolkit (DFTK)^50^, a flexible DFT code written in the Julia programming language. These efforts were greatly facilitated by the ongoing developments in the Julia community towards powerful AD tools^26,27,51–54^. Moreover, DFTK’s simple and tractable code base of only about 10,000 lines of Julia code enabled us to readily equip this existing DFT package with AD capabilities. This is in contrast to previous differentiable DFT software, which was either written from scratch for the purpose to be differentiable or represents a hard fork of an existing code base. We not only believe this integrated development model to be more sustainable in the long run, but we could already benefit from it during this research: recent algorithmic advances on solving DFPT problems^11,55^, which were developed using DFTK, were immediately available to us.

The remainder of this manuscript is structured as follows: We first provide an overview of the key developments required to obtain an AD-based framework for end-to-end differentiable DFT workflows. We then provide six examples to illustrate novel research avenues enabled by the framework, namely: (1) Computation of elastic constants by applying AD on top of AD, (2) engineering a semiconductor band gap, (3) learning XC parameters, (4) optimizing PSPs, (5) propagating the DFT model error to relaxed geometries, and (6) estimating the error in DFT forces due to the chosen plane-wave cutoff. Collectively, these highlight how end-to-end differentiation capabilities turn derivative information into a first-class asset enhancing accuracy, design, and reliability in materials modeling.

## Results

### Algorithmic differentiation framework for plane-wave DFT

**Fig. 2:**
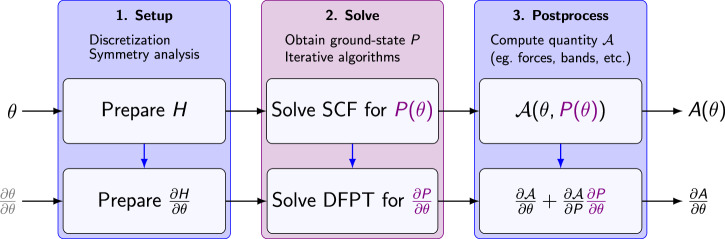
End-to-end derivatives in our AD-DFPT framework. We embed plane-wave DFT into a general-purpose AD system, which across the entire simulation workflow *A* (top row) computes the end-to-end derivative $\frac{\partial A}{\partial \theta }$ (bottom row). Based on forward-mode AD, the full derivative is accumulated starting from the input $\frac{\partial \theta }{\partial \theta }=1$ and following each primitive computational step in order. Here, blue arrows indicate dependencies on intermediate quantities. The AD system automatically obtains the Hamiltonian perturbation $\frac{\partial H}{\partial \theta }$ entering DFPT, as well as the contributions of the postprocessing. For the SCF algorithm (4) we manually define its derivative as the matching DFPT algorithm (5), see details in the main text.

An end-to-end differentiable DFT workflow is achieved by making a DFT code interact seamlessly with a general-purpose AD system. We illustrate our approach using Fig. 2, based on the three conceptual workflow stages: setup, solve, and postprocess.

Entering the setup stage are the simulation parameters *θ* (first row of Fig. 2). Depending on context *θ* may indicate XC functional coefficients, the parameters of the PSP model or the system’s geometry. These parameters are needed for the construction of the plane-wave basis, PSP projectors, and potentials. Ultimately *θ* thus defines the discretized Kohn-Sham Hamiltonian *H*(*θ*, *P*) and energy functional ${\mathcal{E}}(\theta ,P)$ as functions of a trial density matrix *P*. Note that in this section we employ a formulation based on density matrices (instead of explicitly denoting ground-state density, orbitals and occupations) for conciseness; in our calculations equivalent orbital-based representations are used instead (see “Methods”). In the solve stage, the discretized Hamiltonian and energy functional are used to determine the ground state density matrix *P*(*θ*) by iteratively solving the self-consistent field (SCF) equations. Finally, the postprocessing stage evaluates the desired physical quantities, such as total energy, forces, or band structure, which we indicate by the function ${\mathcal{A}}$. Evaluating these quantities ${\mathcal{A}}$ in turn consumes the self-consistent density matrix *P*(*θ*), but may also feature an explicit dependency on simulation parameters *θ*. Considering the workflow in its entirety, from the input parameters to the predicted DFT quantity, thus defines a function

1 $$A(\theta )={\mathcal{A}}\left(\theta ,P(\theta )\right).$$

The end-to-end derivative of this function follows from the chain rule

2 $$\frac{\partial A}{\partial \theta }=\frac{\partial {\mathcal{A}}}{\partial \theta }+\frac{\partial {\mathcal{A}}}{\partial P}\frac{\partial P}{\partial \theta }.$$

Here, the first term represents the explicit dependence of the quantity *A* on the parameters, while the second captures the implicit dependence through the ground-state response $\frac{\partial P}{\partial \theta }$. In particular the implicit term is computationally more involved since it requires differentiating the SCF solution itself.

End-to-end derivatives such as Eq. (2) can be obtained automatically using modern AD systems. In a nutshell this is achieved by working directly on the level of the computer program implementing the workflow *A*, combining three ingredients: (1) A library of known differentiation rules for a set of primitive operations. Such primitives may range from fine-grained operations, e.g., floating-point operation on numbers or matrix arithmetic, to coarse-grained standard algorithms, such as FFTs or eigenvalue solvers. (2) A mechanism to accumulate the full gradient $\frac{\partial A}{\partial \theta }$ from the derivatives of the primitives. Here, the AD system decomposes the entire workflow *A* into a sequence of primitives, applies the tabulated rule to each, and assembles the full gradient via the chain rule. (3) A mechanism for defining new primitives, allowing developers to incorporate domain-specific knowledge into custom differentiation rules.

In our AD-DFPT approach, we use these ingredients to automatically compute the derivative $\frac{\partial A}{\partial \theta }$ by letting the AD system pass through the setup, solve and postprocess stages of the computation, see second row of Fig. 2. The setup stage is treated entirely using the system’s library of differentiation rules as well as its accumulation mechanism. This results in the derivative of the discretized Kohn-Sham Hamiltonian $\frac{\partial H}{\partial \theta }$. Following the workflow, the AD system encounters the solve stage. This stage we define as a custom primitive *imposing* the derivative of the SCF solver to be evaluated by solving a matching linear-response (DFPT) problem. As explained below this yields the response $\frac{\partial P}{\partial \theta }$ from the perturbation $\frac{\partial H}{\partial \theta }$. Finally, we once again let the AD system differentiate through the postprocess stage, assembling the desired derivative as an end result.

Compared to traditional DFPT approaches, AD-DFPT still relies crucially on a general-purpose linear-response (DFPT) solver, requiring manual implementation and careful tuning. However, once this solver is available, the tedious and error-prone hand-derivation of all possible setup and postprocessing combinations (compare Fig. 1) is now completely automated.

To conclude this section, we discuss the custom rule of the solve stage, that is how to compute $\frac{\partial P}{\partial \theta }$ from $\frac{\partial H}{\partial \theta }$. For notational simplicity we suppress spin and Brillouin zone sampling in this discussion and all operators are understood after discretization in a plane-wave basis of size *N*_*b*_. In the solve stage we determine the ground state *P*(*θ*) by minimizing the free energy ${\mathcal{E}}$ including the internal Kohn-Sham energy, the electronic smearing entropy contribution, and the ion-ion electrostatic energy. Usually this is done by satisfying its first-order stationarity conditions, the SCF equations

3 $$\left\{\begin{array}{ll}H(\theta ,P){\psi }_{n}={\varepsilon }_{n}{\psi }_{n},\qquad n=1,\ldots ,{N}_{b},\\ P=\mathop{\sum }\limits_{n=1}^{{N}_{b}}f({\varepsilon }_{n}){\psi }_{n}{\psi }_{n}^{\dagger },\end{array}\right.$$

where the (*ε*_*n*_, *ψ*_*n*_) are *N*_*b*_ orthonormalized eigenpairs of the Hamiltonian and *f* is a smearing function enforcing the correct electron count in *P*. Viewing *f* as a matrix function acting on the Hamiltonian, we can equivalently write (3) as

4 $$P=f\left(H(\theta ,P)\right).$$

From here, the SCF custom rule is obtained naturally by differentiating with respect to *θ*. After rearrangement, this yields

5 $$\frac{\partial P}{\partial \theta }={\left(1-{\chi }_{0}K\right)}^{-1}{\chi }_{0}\frac{\partial H}{\partial \theta },$$

where we use the shorthand ${\chi }_{0}:= \frac{\partial f}{\partial H}$ and $K:= \frac{\partial H}{\partial P}$. Here *χ*_0_ captures the independent susceptibility of the density matrix to a change in the Hamiltonian, while *K* collects the dependence of the Hamiltonian on the density matrix through the nonlinear Hartree and XC terms. Equation (5) is thus a matrix-based formulation of the Dyson equation to compute the interacting response in DFPT. It provides us exactly with the linear response problem to be solved as part of the custom rule of the solve stage.

While the notation of (5) is compact, it involves as principal unknowns density matrices of size ${\mathcal{O}}({N}_{b}^{2})$, which cannot be stored explicitly given the typical number *N*_*b*_ of basis functions used in plane-wave calculations. As common in plane-wave DFT we thus rely on matrix-free formulations and iterative solvers to solve the DFPT problem (5) as described in more details in the Methods section *Orbital-based representation for response*.

### Elasticity: accurate standard properties with minimal human effort

Elastic constants, which characterize the linear response of a material to strain, are fundamental for predicting mechanical stability, sound velocities, and thermomechanical behavior. Traditionally, they are computed either by finite differences (FD)^18,19^ or in the form of providing manual extensions on top of DFPT. While FD methods require carefully tuned strain increments to balance numerical noise and nonlinear effects, DFPT-based approaches involve significant implementation efforts^5,7^, some of which are highly specific to the elastic constants case.

Using our general AD-DFPT framework, elastic constants emerge naturally and with minimal coding effort. The starting point is the stress in Voigt notation, defined from the first derivative of the total energy $E(\eta )={\mathcal{E}}(\eta ,P(\eta ))$ with respect to an applied Voigt strain *η*:

6 $$\sigma (\eta )=\frac{1}{V(\eta )}\frac{\partial {\mathcal{E}}}{\partial \eta },$$

where *V*(*η*) is the volume of the strained unit cell, and we have used the Hellmann–Feynman theorem. Minimizing the total energy, the equilibrium strain *η*^⋆^ induces zero stress. At equilibrium, the elastic stiffness tensor is defined as

7 $$C={\left.\frac{\partial \sigma }{\partial \eta }\right|}_{{\eta }^{\star }}.$$

In DFTK, Hellmann–Feynman stresses are obtained in the postprocessing stage by implementing literally Eq. (6), directly as a call to the AD system, see [^56^src/postprocess/stresses.jl]. With our AD-DFPT framework elastic constants follow immediately from a second invocation of the AD system, computing the end-to-end derivative of the stress *σ* by literally implementing (7). No hand-coded second derivatives are required.

**Fig. 3:**
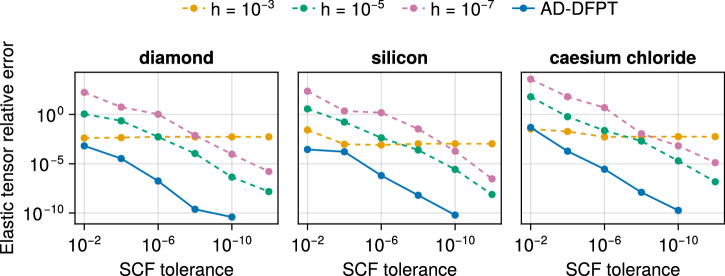
Elasticity. Relative error in the clamped-ion elastic tensor (∥*C* − *C*_ref_∥_*F*_/∥*C*_ref_∥_*F*_) for indicated solids as a function of SCF tolerance. The dashed curves correspond to finite-difference values obtained on top of stresses with step sizes *h* as indicated in the legend. AD-DFPT (solid curve) denotes a direct computation of second-order energy derivatives within our AD framework. All relative errors are computed with respect to the AD-DFPT result at SCF tolerance 10^−12^, see Supplementary Table 1 for the numerical values. AD-DFPT proves to agree well with finite differences at tightest SCF tolerances and be the most precise at looser tolerances, while finite-difference results deteriorate notably for looser SCF tolerance and are sensitive to the step size parameter *h*.

As shown in Fig. 3, our approach inherits the robustness and precision of DFPT. For three solids spanning a range of mechanical hardness, the precision of elastic constants is benchmarked as a function of SCF convergence tolerance. At the tighest tolerance, AD-DFPT agrees well with finite differences, see Supplementary Table 1 for the numerical values. At the loosest SCF tolerances considered, our AD-DFPT approach proves to be the most precise. In contrast, finite-difference results show an increased dependence on SCF tolerances: large steps lead to non-convergent error curves as other error sources dominate, while very small steps amplify the SCF noise and degrade precision. This makes AD-DFPT techniques the preferred approach when considering the trade-off between human implementation time and derivative accuracy.

### Inverse materials design

A successful approach in computational materials discovery is inverse materials design. In contrast to the usual, forward direction to estimate the functional properties of a material given its structure, this approach does the reverse: starting from a desired set of properties it seeks those atomistic structures satisfying these properties most closely^57–59^. For example, when fine-tuning carrier mobilities or lifetimes in semiconductor devices one usually seeks materials with specific band characteristics, e.g., particular band gaps or band valleys. To illustrate how a differentiable DFT code can be beneficial in this endeavor we will consider a toy example, namely the fine tuning of the band gap of bulk gallium arsenide (GaAs) by applying a bulk volumetric strain *η*. This example is inspired by the remarkable successes of strain engineering in the context of proposing better-suited optoelectronic devices^60–63^.

**Fig. 4:**
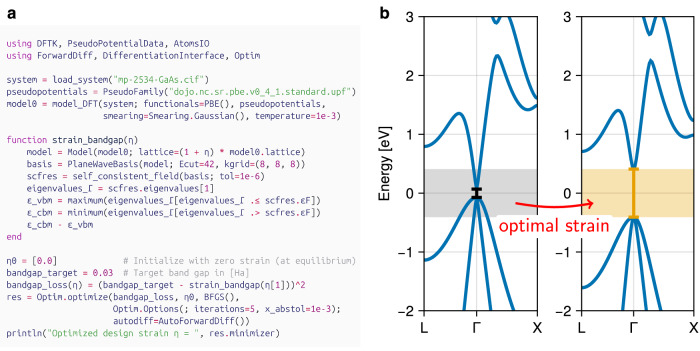
Inverse materials design. **a** Minimal code example tuning the band gap of bulk GaAs with respect to volumetric strain. **b** Band structure of GaAs with the band gap before (left) and after (right) minimizing *L*_bandgap_. Energies are shown relative to the middle of the band gap. The optimizer internally invokes automatic differentiation to compute the required gradient, without requiring user intervention.

Mathematically, we can formulate this problem as the minimization of a loss function such as

8 $${L}_{{\rm{bandgap}}}(\eta )={\left({E}_{g}^{{\rm{target}}}-{E}_{g}(\eta )\right)}^{2},$$

which measures the discrepancy of a predicted band gap *E*_*g*_(*η*) under strain *η* from the targeted value ${E}_{g}^{\,\text{target}\,}$. In our example we will simply obtain *E*_*g*_(*η*) using a Perdew–Burke–Ernzerhof (PBE)^64^ calculation on strained GaAs, see the function strain_bandgap in Fig. 4a. In traditional DFT codes, obtaining the gradient of *E*_*g*_ and thus the gradient of *L*_bandgap_ is challenging, such that naive grid search techniques or other derivative-free methods are still commonly employed — making inverse design problems an expensive endeavor in general.

However, employing an end-to-end differentiable DFT code such as DFTK enables to compute the gradient ∂*L*_bandgap_/∂*η* directly, such that we can use a classic algorithm like Broyden–Fletcher–Goldfarb–Shanno (BFGS)^65^ to rapidly minimize the loss. Figure 4 demonstrates this on our GaAs example, where the optimization achieves the target band gap in just three BFGS iterations. For completeness, we also provide the full user code required to obtain this result in Fig. 4a. Notably, in this example the optimizer transparently triggers the AD-based computation of the required gradient of the bandgap_loss function, thus enabling even novice users to perform gradient-based inverse design with minimal boilerplate.

### Learning the exchange-correlation functional

The data-driven construction of XC functionals has a long history, ranging from early semi-empirical fits of functionals to reference data to more recently increasingly sophisticated machine learning (ML) strategies^45,46,66–75^. Considering materials modeling, ML approaches have considered fitting to reference data such as atomization energies^66,76–78^, lattice constants^79^ and band gaps^71,80^. In most cases, these fitting procedures are indirect: functional parameters are optimized while keeping the electronic density fixed to the density of a baseline functional, such as PBE. Moreover, equilibrium properties are often approximated from energy-volume curve fitting around fixed pre-relaxed structures. These approximations are motivated by cost, but they obscure how parameter changes propagate through the full simulation pipeline, leading to possibly suboptimal fits.

Our differentiable DFT framework enables fully self-consistent, gradient-based optimization of XC parameters against bulk observables, treating both the electronic ground state and the relaxed geometry as differentiable functions of the functional parameters *θ*. For example, to fit lattice constants, we define a loss function over a dataset of materials:

9 $${L}_{{\rm{xc}}}(\theta )=\frac{1}{N}\mathop{\sum }\limits_{i = 1}^{N}{\left(\frac{{a}^{\star }({x}_{i},\theta )-{a}_{i}^{{\rm{expt}}}}{{a}_{i}^{{\rm{expt}}}}\right)}^{2},$$

10 $${\text{where}}\,\quad {a}^{\star }({x}_{i},\theta )=\arg \mathop{\min }\limits_{a}\ E({x}_{i},\theta ,a),$$

11 $$E({x}_{i},\theta ,a)=\mathop{\min }\limits_{P}\ {\mathcal{E}}({x}_{i},\theta ,a,P),$$

${a}_{i}^{\,\text{expt}\,}$ is the experimental lattice constant and *a*^⋆^(*x*_*i*_, *θ*) is obtained by geometry optimization for material *x*_*i*_ at XC parameters *θ*. This setup involves two levels of implicit differentiation: one for the SCF solution and one for the geometry optimization. To handle the latter, we wrap the lattice optimization in a custom differentiation rule that applies implicit differentiation to the optimality condition. This mirrors how we imposed DFPT as the derivative of the SCF solution: internally, the chain rule propagates through stress evaluation, which triggers the DFPT response. The required implicit derivative of the equilibrium lattice constant with respect to functional parameters takes the form

12 $$\frac{\partial {a}^{\star }}{\partial \theta }=-{\left(\frac{{\partial }^{2}E}{\partial {a}^{2}}\right)}^{-1}\left(\frac{{\partial }^{2}E}{\partial \theta \partial a}\right)$$

evaluated at the converged structure. Using this expression, the full loss gradient $\frac{\partial {L}_{{\rm{xc}}}}{\partial \theta }$ is composed automatically within our framework.

**Fig. 5:**
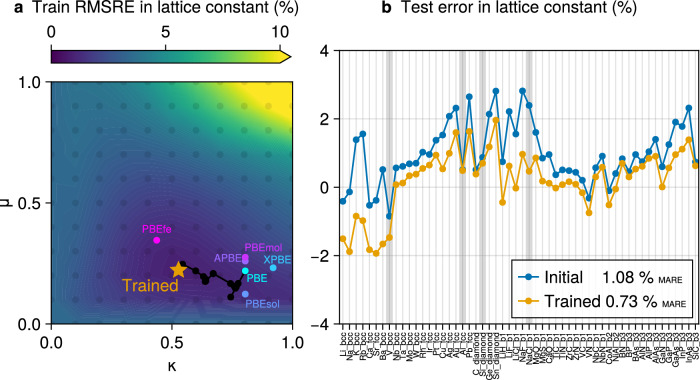
Learning the exchange-correlation functional. Experimental lattice constants are targeted for solids in the Sol58LC dataset^81^. **a** The training loss landscape in two parameters *μ*, *κ* of the PBE functional^64^ is visualized by an exhaustive grid search of the root mean squared relative error (RMSRE), along with several variants from the literature^82,108–111^. The efficient trajectory of the AD-DFPT-enabled optimization is shown in black. **b** Relative lattice constant errors for solids in the test set. The train set of Si, Al, V, NaCl is indicated in gray. Fine-tuning improves agreement on average across the dataset, though some metals (e.g., Li, Na, and even V) show overcompensation.

In Fig. 5, we start from the PBE functional and optimize two of its parameters (see details in the Methods section) on four lattice constants selected from the Sol58LC benchmark set^81^. The fine-tuned functional reduces the root mean squared relative error compared to several standard PBE variants, with all predictions obtained through fully self-consistent calculations. Some metals exhibit overcompensation, including vanadium even though it was included in training. The optimizer has to balance competing regimes, reflecting the limited flexibility of the chosen parametrization. Such trade-offs are well established in the construction of semilocal functionals, for instance between the accuracy of PBE for molecules and that of PBEsol for solids^82^, both generalized gradient approximations (GGA). We remark that such trade-offs can be improved by using more expressive forms such as meta-GGAs^79,83,84^, which is however beyond the scope of this work. Overall, this example illustrates how AD enables systematic, targeted exploration of functional refinements in a fully self-consistent setting.

### Property-driven pseudopotential optimization

PSPs are essential for the efficiency of plane-wave DFT, yet they can introduce errors of similar magnitude to that of the XC functional^85^. Such PSP errors can become especially large if a PSP is employed in combination with a functional, that differs from the functional used to fit the PSP parameters in the first place^86^. Despite ongoing efforts to automate validation and benchmarking^85,87,88^, the PSP fitting process itself remains largely manual or based on derivative-free optimization^89–92^.

Here, our AD-DFPT framework provides new opportunities for PSP generation. We demonstrate these briefly using the example of fitting a PSP for lithium, a lightweight element where the choice of PSP strongly affects smoothness and transferability. Specifically, we train a valence-only one-electron PSP to reproduce the energy-volume curves of a more accurate, three-electron semicore PSP across two structurally distinct compounds: elemental Li in the body-centered cubic (BCC) phase and LiO in a rocksalt structure. Note that this semicore potential is only a stand-in to avoid the additional complexity of performing an all-electron calculation.

**Fig. 6:**
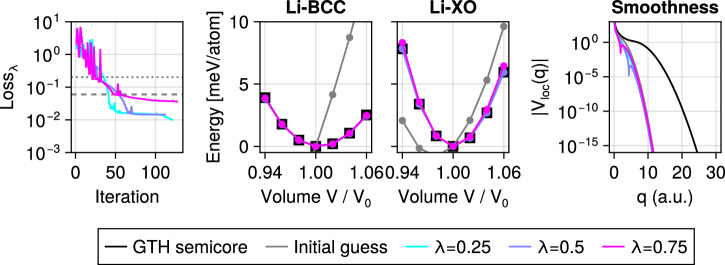
Property-driven pseudopotential optimization. A valence-only pseudopotential for Li is trained against energy-volume curves of the more expensive semicore pseudopotential. The rightmost plot provides the decay of the Fourier components of the resulting local potential *V*_loc_ wrt *q*, the magnitude of the wave vector. The parameter *λ* controls the relative weight of the unary and oxide loss terms as detailed in the Methods section.

Figure 6 summarizes the optimization result: we observe substantial improvement in agreement with the reference, while preserving the smoothness of the potential required for rapid convergence of the discretization. To quantify agreement, we minimize a composite loss function combining normalized energy-volume curve errors across both compounds. Since the loss depends only on total energies, the required gradients with respect to PSP parameters are computed efficiently using the Hellmann-Feynman theorem.

Our AD-DFPT framework thus enables a gradient-based fitting of PSP parameters directly employing bulk DFT observables in our loss function.

### Propagating XC functional uncertainty

In recent years a number of approaches have been developed to provide statistical uncertainty estimates in the parameters of the XC functional. Examples are the Bayesian error estimation functional (BEEF) family^66,76^, approaches based on Bayesian linear regression^78^ or mixtures of established functionals^93^. Provided such an error-aware DFT functional is chosen, the built-in parameter uncertainty estimate ought to be propagated to physical predictions, such as equilibrium geometry and lattice constants. For this purpose, previous work relied on sampling-based ensemble propagation methods, combined with additional approximations such as non-self-consistent calculations and equation of state fitting^66,78,93^.

**Fig. 7:**
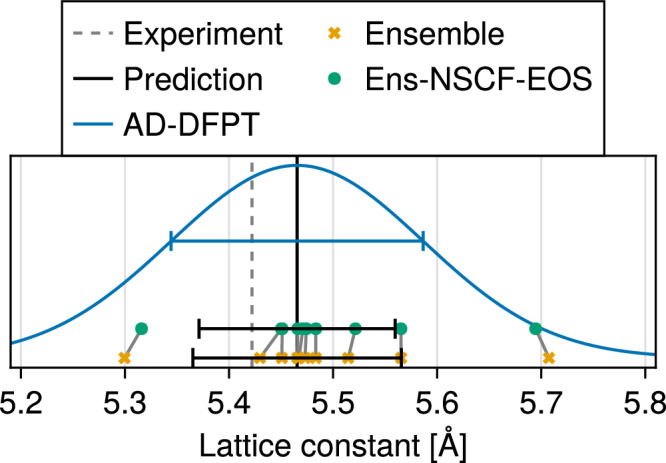
Propagating XC functional uncertainty. Predictive uncertainty in the relaxed lattice constant of silicon obtained by propagating the parameter uncertainty of the BEEF functional^66^ forward using three methods: full ensemble (10 geometry relaxations, orange), an ensemble based on non-self-consistent equation-of-state fits (Ens-NSCF-EOS, green), and a linearized analytic approximation using end-to-end differentiation and AD-DFPT (blue). The latter relies only on a single linearization around the mean parameters, avoiding the need for a choice of ensemble size or further approximations.

The ability to compute end-to-end derivatives in our AD-DFPT framework provides a new ingredient for such uncertainty propagation tasks, namely, to linearize entire computational workflows. We demonstrate this in the blue curve in Fig. 7, which displays the distribution of lattice constants resulting from propagating the uncertainty encoded in the BEEF parameters forward through both the SCF and the geometry optimization.

Specifically, if *a*^⋆^(*θ*) denotes the optimal lattice constant depending on the BEEF parameter value *θ*, a linearization around the mean parameter *θ*_0_ yields

13 $${a}^{\star }(\theta )\approx {a}^{\star }({\theta }_{0})+J\cdot (\theta -{\theta }_{0}).$$

Here, $J=\frac{\partial {a}^{\star }}{\partial \theta }{| }_{{\theta }_{0}}$ is the same total derivative as Eq. (12), readily computed by applying our AD-DFPT framework. Since the BEEF posterior for *θ* is modeled as a Gaussian distribution, applying a linear pushforward approximation again yields an analytic Gaussian ${\mathcal{N}}({a}^{\star }({\theta }_{0}),J \Sigma J^\top)$ for the uncertainty in *a*^⋆^, where *θ*_0_ is the mean and *Σ* the covariance of the BEEF posterior.

Notably, AD-based linearization provides a viable alternative to ensemble propagation methods. In these latter kinds of methods, *N* sets of XC parameters are sampled from the BEEF posterior and each is considered independently. A full ensemble therefore requires *N* separate geometry relaxations, which in practice is prohibitively costly. To reduce cost, previous works^66,78,93^ have often employed an approximate procedure that we denote here as Ens-NSCF-EOS. Instead of re-relaxing the structure for each sample, Ens-NSCF-EOS performs an equation-of-state fit over seven fixed volumes around the mean-parameter equilibrium, using non-self-consistent energy evaluations based on the mean-parameter density and orbitals.

As shown in Fig. 7, our AD-based linearization yields results comparable to an ensemble of 10 independent geometry relaxations (orange) and its Ens-NSCF-EOS approximation (green). Unsurprisingly, the error bars from such small ensembles are not converged. While agreement improves when increasing the ensemble size to 30 (not shown in the figure), this indicates the challenge of selecting an ensemble size, which gives good results, but remains computationally feasible.

In contrast, the AD-DFPT linearization for uncertainty propagation avoids tuning choices such as ensemble size as well as any further approximations by equation-of-state fits or non-self-consistent evaluations. Instead, it requires only a single relaxation and derivative evaluation, and it extends mechanically to any quantity available in the end-to-end differentiable workflow.

### Estimation of the plane-wave basis error

Choosing the kinetic energy cutoff *E*_cut_ for plane-wave computations remains a difficult tradeoff between accuracy and cost with the ideal cutoff depending on the precise system, the computed properties, and the desired accuracy. Recommended cutoffs from PSP libraries cannot always reflect these nuances. At the same time performing explicit convergence studies for each simulation greatly increases the overall cost of the simulation. Without a cheap and precise way to control the discretization error, one often has no other option but to resort to running simulations with an increased *E*_cut_ to ensure convergence. Especially when performing massive data generation over a large dataset of material structures this drives up the total computational cost considerably^94^.

Yet, reliable error estimates have received increasing interest in the mathematical community of DFT. In particular, Cancés et al. have proposed a strategy^95^ to estimate the DFT plane-wave discretization error. This strategy computes an error estimate that is specific to the system and property of interest, making it a promising avenue. Based on a standard SCF and the resulting initial density matrix *P*, the approach results in a perturbative correction *δP* which approximates the plane-wave discretization error in *P*. Here, the key contribution of ref. ^95^ is an efficient algorithm, such that the effect of increasing the basis is approximately captured, but without having to perform the full SCF in a larger basis.

Coupling this approach with our differentiable DFT framework enables the propagation of the error estimate *δP* to quantities of interest such as the interatomic forces *F*. In fact, early versions of our AD framework were already used in ref. ^95^. By linearization of the force computation *F*(*P*), the discretization error estimate in the forces is:

14 $$\delta F\approx \frac{\partial F}{\partial P}\delta P.$$

This quantity is readily computed from *P* and *δP* with a single forward-mode AD pass. Notably, the perturbation *δP* in this equation is computed following^95^, which already involves solving some approximate Dyson equation. Thus, no additional solution of (5) is required as part of the AD procedure as we only use AD to compute $\frac{\partial F}{\partial P}$.

**Fig. 8:**
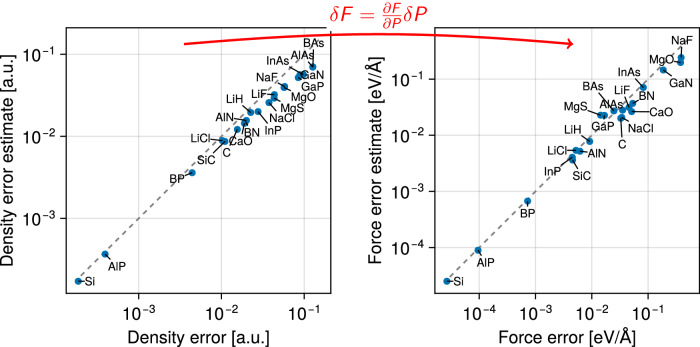
Estimation of the plane-wave basis error. Propagation of plane-wave discretization error estimates from the density to the forces using AD. The estimates, following the method from ref. ^95^, approximate the error in the density and forces due to the low energy cutoff *E*_cut_ of 20 Ha used for all solids. They correlate well with reference values. The density error metric is the integrated absolute density difference over the unit cell, normalized by the number of electrons. The force error metric is the largest force difference magnitude, i.e., $\mathop{\max }\limits_{\,{\text{atom}}\,i}\parallel {{\Delta }}{F}_{i}{\parallel }_{2}$.

We illustrate this technique on a range of 21 insulating bulk solids from the Sol58LC dataset, using a fixed plane-wave kinetic energy cutoff of *E*_cut_ = 20 Ha. Of note, this value is lower than the recommended cutoff for most solids. As such, we expect a large system-dependent discretization error in the converged density and the derived forces, which we would hope to capture with the error estimate procedure. Indeed, as Fig. 8 summarizes, we obtain an excellent correlation between the error estimate and the reference error (obtained by comparing against a high-*E*_cut_ computation). In particular, not only is the electronic density error accurately estimated (left), but so is the error in the force (right), computing using (14).

Hand-implementing unusual derivatives such as $\frac{\partial F}{\partial P}$ is already a tedious and error-prone process for experienced code developers, but can become an insurmountable obstacle for practitioners testing such error-estimation strategies. Our differentiable plane-wave DFT code seamlessly provides such derivatives and in this way furthers the development of error estimation strategies: by computing appropriate derivatives propagating the density error estimates to other quantities of interest becomes readily feasible.

## Discussion

In this article we presented the AD-DFPT framework for DFT gradient computation, an accurate and automated approach to compute end-to-end derivatives across the entire DFT workflow. By integrating AD techniques with classic DFPT, it shares the favorable robustness and accuracy properties of established DFPT-based methods, while extending their reach to arbitrary input parameters and postprocessing quantities. In particular, it avoids common pitfalls of finite-difference techniques, such as the complex interplay between the optimal step size and numerical details such as plane-wave cutoff or SCF tolerance. This combination opens the door to high-precision gradient-based DFT workflows even for researchers who are not DFT implementation experts.

We demonstrated multiple emerging research opportunities, covering a wide range of tasks, such as inverse materials design, uncertainty quantification of DFT simulations, or the learning of improved functionals or PSPs. Importantly, with an AD-DFPT implementation at hand, we were able to push the state of the art and directly target relaxed self-consistent material properties as reference data, instead of relying on surrogate losses or fixed-density approximations. Given the increasing interest in integrating ML approaches directly within the formulation of DFT models, our developments provide an important and timely foundation to support such efforts.

While AD provides a systematic route to differentiation, its application does not eliminate the inherent mathematical and numerical challenges of a physical model as complex as DFT. We emphasize this point on three practical issues. First, for specific expressions, the standard derivative rules of the AD system do not always yield a numerically stable derivative. Such cases can be solved by providing a custom rule with a hand-coded derivative implementation, or preferably by modifying the initial undifferentiated code to enhance stability; see the example of the Fermi-Dirac function in the Supplementary Information. Second, some DFT quantities are not always differentiable. An example is the band gap, since its definition involves minima and maxima over eigenvalues which are non-differentiable at crossings. In such cases, both numerical and AD techniques will still compute *some value* for the derivative without an indication that the numerical result may be unreliable. In this regard, various solutions (e.g., appropriate smoothening of the differentiated function^96^, Chapter 5) have been suggested in the AD community^22^, Chapter 14, which should be explored in the context of AD-DFPT in future work. Third, the crystal symmetry analysis^97^ used by DFT codes to reduce the effective number of *k*-points needs to be adapted to additionally consider crystal perturbations. Indeed, geometric perturbations of a crystal typically break some of its symmetries, which must then be filtered out; see an illustration in Supplementary Fig. 1. An automated algorithm is work in progress.

The current implementation of AD-DFPT in DFTK fully supports forward-mode propagation of general parameter derivatives through DFT computations involving norm-conserving PSPs, GGA functionals and no spin polarization. While considering other models or spin-polarized systems requires additional implementation effort, such extensions integrate well into the presented AD-DFPT framework. In particular, automatically generating the derivatives of the setup and postprocess stages in Fig. 2 is not limited to our current setting and generalizes straightforwardly to other functionals and models. Going beyond GGA functionals, for example, is primarily limited by the capabilities of the underlying DFPT solver. Developing a reliable and efficient DFPT solver capable of treating meta-GGA or hybrid-DFT functionals, remains a considerable technical challenge and poses an important avenue for future research to extend the applicability of AD-DFPT methods. Finally, our forward-mode implementation prepares the ground towards more challenging reverse-mode AD techniques, enabling, as an outlook the efficient optimization in high-dimensional parameter spaces, for example, when training deep-learning XC functionals.

Looking ahead to the nested workflows common in materials simulation, packages for higher-level tasks such as geometry optimization or molecular dynamics could provide their own differentiation rules, relying on the underlying DFT code to compute parameter derivatives transparently. This would achieve a clear separation of concerns and make differentiation of such workflows naturally composable.

In summary, AD-DFPT brings plane-wave DFT in line with the general progress towards differentiable programming in scientific computing. As exemplified, the resulting end-to-end differentiable workflows have the potential to significantly enhance materials design, error control, and systematic learning of model parameters in computational materials science.

## Methods

In the following, we provide details on the implementation of the AD-DFPT framework in DFTK, followed by an outline of the computational setup of each example.

### Orbital-based representation for response

The density-matrix-based formulation of Eq. (5) is convenient to illustrate the mathematical structure of DFPT. However, manipulating objects such as dense Hamiltonians or density matrices (with ${\mathcal{O}}({N}_{b}^{2})$ storage cost) is prohibitive for basis sets with a large number *N*_*b*_ of functions, such as plane-waves. In our computations, we therefore follow the standard approach to employ sparse representations in terms of Kohn–Sham orbitals as well as iterative techniques for solving response problems. We sketch these briefly in this section.

Consider the non-differentiated form of DFPT, that is, the SCF problem (4). To avoid the ${\mathcal{O}}({N}_{b}^{2})$ storage cost of Hamiltonians or density matrices when solving the Kohn–Sham eqs (3) one employs iterative methods. The density matrix is represented implicitly as a truncated set of *N* partially occupied Kohn–Sham orbitals *ψ*_*n*_ and occupations *f*_*n*_ with *N* ≪ *N*_*b*_, thus in ${\mathcal{O}}({N}_{b})$ storage. In tur,n the Hamiltonian is represented as a sum of three sparse terms with again ${\mathcal{O}}({N}_{b})$ storage: a diagonal kinetic term, the low-rank nonlocal PSP projectors, and real-space local potentials applied via FFT-based convolutions^17^.

Taking the derivative of such structured representations retains this structure. Therefore sparse ${\mathcal{O}}({N}_{b})$ representations for the corresponding perturbations *δP* and *δH* can similarly be constructed and carried through all stages of the DFT workflow of Fig. 2. More precisely any Hamiltonian perturbation *δH* can be expressed through perturbations of its sparse terms and any admissible (i.e., representable in the *N*-truncated subspace) density matrix perturbation *δP* can in turn be parametrized by *N* pairs (*δψ*_*n*_, *δf*_*n*_) of perturbations to its orbitals and occupations

15 $$\delta P=\mathop{\sum }\limits_{n = 1}^{N}\delta {f}_{n}{\psi }_{n}{\psi }_{n}^{\dagger }+{f}_{n}\left(\delta {\psi }_{n}{\psi }_{n}^{\dagger }+{\psi }_{n}\delta {\psi }_{n}^{\dagger }\right).$$

We remark that the choice of *δψ*_*n*_ and *δf*_*n*_ to represent *δP* is not unique, which in DFTK is fixed according to the *minimal gauge* described in ref. ^11^.

Based on this orbital-based representation for *δP* the response in Eq. (5) is solved iteratively using the inexact generalized minimal residual (GMRES) algorithm of ref. ^55^, which only requires matrix-vector products of (1 − *χ*_0_*K*) with trial vectors. For the latter each application of *χ*_0_ to a trial Hamiltonian perturbation *δH* computes the orbital responses *δψ*_*n*_ as a sum of two ingredients: a sum-over-states formula for the occupied contributions and the iterative solution of the Sternheimer equations for the unoccupied contributions, using a tailored preconditioned conjugate gradient algorithm^11^. This reflects a broader design principle: iterative algorithms for linearized response problems parallel those used in the underlying nonlinear problem. In our case, the nested iterative structure of Anderson-accelerated SCF iteration with an inner LOBPCG (locally optimal block preconditioned conjugate gradient^98^) eigensolver carries over to a GMRES iteration (the linear analog of Anderson acceleration^99^) with an inner conjugate gradient algorithm for the Sternheimer equations.

### Integration of forward-mode AD within DFTK

For our AD-DFPT framework in DFTK we employ the Julia forward-mode AD package ForwardDiff^26^, which provides AD capabilities using a technique known as operator-overloading AD. In a nutshell, ForwardDiff defines a new number type, the dual number. When used instead of a standard floating-point number, it changes a function’s behavior by computing simultaneously its value and derivatives, thus in line with executing Fig. 2 from left to right in both rows simultaneously. For standard primitive functions (e.g., multiplication, addition, trigonometric functions, LAPACK-based linear algebra^100^), ForwardDiff provides differentiation rules based on such dual numbers as well as appropriate chain rule expressions to compose derivative results.

Relying on Julia’s multiple dispatch mechanism, DFTK is generic in the employed floating-point type, which includes dual numbers. The largest part of the code base is thus made differentiable simply by switching the floating-point type from normal floating-point numbers to dual numbers when the gradient of a DFT quantity should be computed. The only exceptions are cases where a custom rule should be employed for differentiation instead of a decomposition down to standard primitives.

In ForwardDiff such custom rules can be provided by overloading a function with a method that is specialized to arguments of the dual number type. On top of our standard self_consistent_field function to execute the first row of the solve stage of Fig. 2 we therefore also provide a special self_consistent_field function for dual numbers in DFTK. Whenever ForwardDiff attempts to compute the derivative of the solve stage then this function in DFTK will be called, enabling us to extract $\frac{\partial H}{\partial \theta }$ from the AD system, solve the Dyson Eq. (5) using our DFPT solver and re-inject the solution $\frac{\partial P}{\partial \theta }$ back to ForwardDiff.

### Elasticity: accurate standard properties with minimal human effort

For all calculations we employ the PBE functional^64^, PseudoDojo PSPs^91^, Gaussian smearing of 10^−3^ Ha and a 8 × 8 × 8 *k*-mesh. The plane-wave cutoff was chosen following the *normal* recommendations^91^. Crystal structures for diamond, silicon (diamond structure), and cesium chloride are generated from the Atomic Simulation Environment^101^, and are relaxed before computing elastic constants. For the computation of the elasticity tensor only a single strain pattern of *η* = (1, 0, 0, 1, 0, 0) has been used, which recovers all (*C*_11_, *C*_12_, *C*_12_, *C*_44_, 0, 0) in our cubic crystals. Any crystal symmetries of the unstrained crystal, which would be broken by this perturbation, are removed during all computations. For the finite-difference computations, we employ a central formula. The SCF tolerance corresponds to the *L*_2_ error in the density.

### Inverse materials design

The fully self-contained code for the inverse design example is included in Fig. 4.

### Learning the exchange-correlation functional

In this example we optimize the two free parameters *κ* and *μ* in the PBE exchange enhancement factor^64^

16 $${F}_{X}(s)=1+\kappa -\kappa /(1+\mu {s}^{2})$$

where *s* is the reduced density gradient. The reference experimental lattice constants, including zero-point corrections are taken from the Sol58LC data set^81^. The outermost parameter optimization loop to minimize Eq. (11) employs BFGS as implemented in ref. ^102^ with a backtracking linesearch and implicit parameter gradients derived according to Eq. (12). DFT calculations use a Gaussian smearing of 10^−3^ Ha and PseudoDojo PSPs^91^. We follow the *normal* recommendations^91^ for the plane-wave basis cutoffs, giving a range from 18 to 49 Ha. Additionally, kinetic energy cutoff smearing is used^103^ to enforce a smooth lattice relaxation. Note that the *k*-grid, ensuring a maximal *k*-point spacing of 0.15 Å^−1^, is determined once for each solid at the initial step of the optimization and held fixed afterwards.

### Property-driven pseudopotential optimization

We optimize six selected parameters of a valence-only Li PSP in the Goedecker–Teter–Hutter (GTH) parametrization^104^. The initial guess is the “LDA” version, while the semicore reference is from the PBE table^15,105^. Reference structures of Li-BCC and LiO rocksalt ("Li-XO") are taken from ref. ^85^, corresponding to the relaxed all-electron PBE structures. For each compound, we generate energy-volume curves using seven uniformly spaced volumes in the range ±6% around the reference. We impose a normalized energy-volume loss function per compound, adapted from the recent *ε*-metric^85^:

17 $$\varepsilon =\sqrt{\frac{{\sum }_{i}{({E}_{i}^{a}-{E}_{i}^{b})}^{2}}{\sqrt{{\sum }_{i}{({E}_{i}^{a}-\overline{{E}^{a}})}^{2}{\sum }_{i}{({E}_{i}^{b}-\overline{{E}^{b}})}^{2}}}}.$$

Here, ${E}_{i}^{a}={E}^{a}({V}_{i})$ and ${E}_{i}^{b}={E}_{b}({V}_{i})$ are total energy differences relative to the energy at the reference volume *V*_0_, at volume *V*_*i*_ from models *a* and *b*, respectively, and $\overline{{E}^{a}},\overline{{E}^{b}}$ are their volume-averaged energies. The overall training loss is defined as a weighted sum over the two compounds as *L*_pseudo_(*θ*) = *λε*_Li-BCC_(*θ*) + (1 − *λ*)*ε*_LiO_(*θ*), where *θ* now denote the PSP parameters and *λ* controls the balance between the two training structures. The outermost parameter optimization loop uses BFGS as implemented in ref. ^102^ with a backtracking linesearch and total energy parameter gradients computed using the Hellmann–Feynman theorem. DFT calculations use Gaussian smearing of 0.00225 Ha with a moderate 8 × 8 × 8 *k*-mesh consistently for both reference and prediction, comparable to recent PSP benchmarks^88,91,106^. The plane-wave basis cutoff used is 120 Ha for semicore Li and O, and 20 Ha for valence-only Li.

### Propagating XC functional uncertainty

Numerical parameters are identical to the example *Learning the exchange-correlation functional*, except for the choice of DFT functional. Here, we employ the BEEF XC functional with the parameters reported in ref. ^66^. The silicon geometry used has been optimized tightly using the mean XC parameters before considering the uncertainty propagation. For the Ens-NSCF-EOS ensemble, total energies are recomputed on a fixed grid of seven volumes symmetrically spaced within ±6% around the equilibrium volume, using the mean-parameter density and orbitals. The equilibrium lattice constant for each sampled parameter is then extracted by fitting a Birch–Murnaghan equation of state. For the AD-based linear pushforward, the derivative of the relaxed lattice constant with respect to the XC parameters is computed via implicit differentiation, using a single geometry optimization and derivative computation at the mean parameters.

### Estimation of the plane-wave basis error

We use the PBE functional^64^, the PseudoDojo PSPs, a uniform plane-wave cutoff of 20 Ha and a minimal *k*-spacing of 0.15 Å^−1^. For each system, the first atom is displaced compared to the equilibrium structure such that the largest interatomic force magnitude is around 0.5 eV Å^−1^. The error estimates are computed following^95^ using a reference cutoff *E*_cut,ref_ = 1.5 × the *high* recommendation for the PSPs^91^. The reference error is obtained by comparison against an expensive reference SCF computation for each system, using *E*_cut,ref_ as the plane-wave cutoff and otherwise the same parameters.

## Supplementary information

Supplementary information

## Data Availability

All data necessary to reproduce the experiments and plots are included in the code repository.

## Code availability

Our AD-DFPT framework is directly included in DFTK v0.7.16 at https://dftk.org. An archived copy is available at ref. ^56^. All code to reproduce calculations and plots is included in the public repository at https://github.com/niklasschmitz/ad-dfpt, archived at ref. ^107^.
