# Supplementary material for: Algorithmic differentiation for plane-wave DFT: materials design, error control and learning model parameters
Source: NPJ Comput Mater. 2025 Dec 4;12(1):6. doi: 10.1038/s41524-025-01880-3 (PMC12774857; doi:10.1038/s41524-025-01880-3)
Supplement: Supplementary file 1 — Supplementary information [file 41524_2025_1880_MOESM1_ESM.pdf]

Supplementary information for "Algorithmic differentiation for plane-wave DFT: materials design, error control and learning model parameters"

Niklas Frederik Schmitz,[1, 2, *] Bruno Ploumhans,[1, 2] and Michael F. Herbst[1, 2, †]

[1] *Mathematics for Materials Modelling (MatMat), Institute of Mathematics & Institute of Materials, École Polytechnique Fédérale de Lausanne, 1015 Lausanne, Switzerland*

[2] *National Centre for Computational Design and Discovery of Novel Materials (MARVEL), École Polytechnique Fédérale de Lausanne, 1015 Lausanne, Switzerland*

Elastic constants

Complementing Figure 3 of the main text, Supplementary Table I contains the computed clamped-ion elastic constants for the tightest used SCF tolerance 10^{-12}. The table demonstrates an excellent agreement of 7–8 digits between our AD-DFPT approach and finite differences. In Figure 3 we therefore use the AD-DFPT results of Supplementary Table I as the reference values for our error computation. However, we remark that due to the tight agreement all relative errors larger than 10^{-7} are to leading order unchanged if the finite difference values were used as the reference. In particular none of the finite difference curves shown in Figure 3 would look visually different if the accurate finite-difference result from Supplementary Table I was employed as the reference.

To obtain this data we employed the same computational parameters as described in the Methods section. These settings (e.g. the k-mesh) have been chosen to keep the computational cost small when comparing to finite differences, and do not provide a fully converged result.

	**diamond**		
	C_{11}	C_{12}	C_{44}
AD-DFPT	**1056.8864**147	**125.1813**098	**564.6239**727
FD	**1056.8864**246	**125.1813**203	**564.6239**743
MP [1]	1053	126	561
	**silicon**		
	C_{11}	C_{12}	C_{44}
AD-DFPT	**153.0503**755	**56.44207**2822	**99.72657**670
FD	**153.0503**761	**56.44207**3688	**99.72657**734
MP	153	57	74
	**caesium chloride**		
	C_{11}	C_{12}	C_{44}
AD-DFPT	**32.60613**704	**5.50990**2647	**4.97546**906
FD	**32.60613**226	**5.50990**3687	**4.97546**917
MP	33	6	5

Supplementary Table I. **Elasticity.** Computed PBE clamped-ion elastic constants C_{ij} (GPa) from our AD-DFPT framework versus finite difference (FD) computation of the stress (step size $h = 10^{-5}$). Elastic constants from the Materials Project (MP) database [1] are shown for additional context. Note that unlike our computations, the MP quantities employ PAW potentials as well as ionic relaxations, explaining the large discrepancy in C_{44} for silicon [2].

Derivative instability: Fermi-Dirac example

As highlighted in the Discussion, the standard rules of the AD system can lead to numerically unstable derivative computations. To restore stability, a custom rule can be defined, but the preferable solution is to switch to another mathematically equivalent expression. In this section, we illustrate this point

on the Fermi-Dirac function:

$$f_{\mathrm{FD}}(x) = \frac{1}{1+e^x}. \tag{S01}$$

An AD system will differentiate f_{FD} to

$$f'_{\mathrm{FD}}(x) = \frac{e^x}{(1+e^x)^2}. \tag{S02}$$

For large x (and overflowing exponential) this leads to the floating-point operation Inf/Inf = NaN, instead of the expected answer $f'_{\mathrm{FD}}(\infty) = 0$.

To circumvent this problem in DFTK we employ the equivalent expression

$$f_{\mathrm{FD}}(x) = \frac{e^{-x}}{e^{-x}+1} \qquad \text{for } x > 0 \tag{S03}$$

whenever x is positive (while we keep the original expression (S01) for $x \leq 0$). The AD-computed derivative of (S03) becomes

$$f'_{\mathrm{FD}}(x) = \frac{-e^{-x}}{(e^{-x}+1)^2}, \tag{S04}$$

which still features an underflow of e^{-x} for large x. However, this time the final result in finite-precision arithmetic will remain the correct answer 0.

Symmetry-breaking crystal perturbations

In the Discussion, we mentioned that symmetry analysis must account for crystal perturbations. Consider a conventional diamond silicon unit cell, and let us compute the response of the electronic density wrt. the displacement of a single atom along the x direction. This perturbation breaks some symmetries such as 90° rotation around the silicon atom situated in the xy plane.

Supplementary Figure 1 compares three approaches to compute this derivative. The first (leftmost) approach uses finite differences. For each of the two SCF computations, the symmetry analysis is performed separately on the respective input structures, and will thus disregard any broken perturbation. The second approach is AD-DFPT with automatically detected symmetries. In our current implementation, automatic symmetry analysis is performed by Spglib [3] using only the *unperturbed* lattice parameters and atom positions; these symmetries are then used for both the SCF and AD-DFPT computations. While simple, this naive approach leads to over-symmetrization of the density response, leading to a result in qualitative disagreement with finite differences. The third (rightmost) approach is AD-DFPT with all symmetries disabled. This avoids erroneous over-symmetrization and provides the expected agreement with finite differences, but comes with additional computational cost.

For maximal efficiency, AD-DFPT computations should be performed with the symmetry group of the perturbed crystal. Determining this symmetry group can be done in two practical alternative ways. First, the symmetry group computed from a finite perturbation of the lattice and atoms positions with a small step size can be passed to our setup. We used this simple approach for the efficient implementation of elastic constants, which indeed require symmetry-breaking lattice strains. Second, one may avoid this extra step and directly perform a symmetry analysis of the perturbation components inside of a custom differentiation rule. An implementation of this second approach would make the first approach obsolete and is currently work in progress.

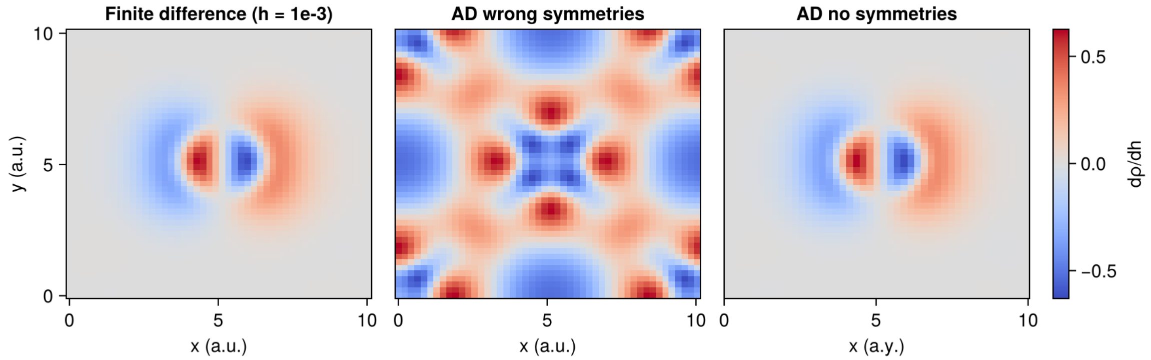

Supplementary Figure 1. **Symmetry-breaking perturbation**. Derivative of the electronic density wrt. the displacement of an atom along the x direction. This perturbation breaks some symmetries of the diamond silicon crystal. Finite differences as well as symmetry-disabled AD-DFPT agree qualitatively, whereas AD-DFPT with naive symmetry analysis leads to an over-symmetrized density response. The density derivatives are shown in the xy plane that contains the moved silicon atom.

* niklas.schmitz@epfl.ch
† michael.herbst@epfl.ch

[1] M. de Jong, W. Chen, T. Angsten, A. Jain, R. Notestine, A. Gamst, M. Sluiter, C. Krishna Ande, S. van der Zwaag, J. J. Plata, C. Toher, S. Curtarolo, G. Ceder, K. A. Persson, and M. Asta, Charting the complete elastic properties of inorganic crystalline compounds, Scientific Data **2**, 150009 (2015).

[2] C. Lin, S. Poncé, F. Macheda, F. Mauri, and N. Marzari, Elastic constants and bending rigidities from long-wavelength perturbation expansions (2024), arXiv:2412.18482 [cond-mat.mtrl-sci].

[3] A. Togo, K. Shinohara, and I. Tanaka, Spglib: a software library for crystal symmetry search, Science and Technology of Advanced Materials: Methods **4**, 2384822 (2024).
